# Factors associated with ‘honour killing’ in Afghanistan and the occupied Palestinian Territories: Two cross-sectional studies

**DOI:** 10.1371/journal.pone.0219125

**Published:** 2019-08-08

**Authors:** Andrew Gibbs, Nader Said, Julienne Corboz, Rachel Jewkes

**Affiliations:** 1 Gender and Health Research Unit, South African Medical Research Council, Pretoria, South Africa; 2 Centre for Rural Health, School of Nursing and Public Health, University of KwaZulu-Natal, Durban, South Africa; 3 Arab World for Research and Development (AWRAD), Ramallah, Palestine; 4 Independent Consultant, Kabul, Afghanistan; 5 School of Public Health, University of Witwatersrand, Johannesburg, South Africa; Stellenbosch University, SOUTH AFRICA

## Abstract

‘Honour killing’, the murder of women to preserve family reputation, is well recognised but infrequently systematically researched. This paper has three hypotheses. First, in families where women report an ‘honour killing’ there is more violence against women and girls, second these women are more likely to report more patriarchal gender attitudes than others, and third these families are exposed to higher levels of poverty. We asked (n = 1461) women enrolled in a trial in Afghanistan, and (n = 535) in a population-based sample in the occupied Palestinian Territories (oPT) if there had ever been an ‘honour killing’ in their family. In Afghanistan, 2.3% (n = 33), and the oPT 7.7% (n = 41), reported this. We built separate multivariable logistic regression models for each country, and for married and unmarried women in each country. Among Afghan married women, ‘honour killing’ was associated with borrowing because of hunger (adjusted odds ratio [aOR]8.71, 95%CI 2.27–33.40), easier access to money in emergency (aOR11.39, 95%CI 3.05–42.50), and violence within the family; intimate partner violence (IPV) (aOR3.73, 95%CI 1.12–12.36), and IPV and mother-in-law violence (aOR10.52, 2.60–42.56). For unmarried women in Afghanistan, ‘honour killing’ was associated with easier access money in an emergency (aOR4.06, 95%CI 0.85–19.37), household violence (hit by parent or sibling, or parent and sibling [aOR5.47, 95%CI 0.82–36.70; aOR7.37, 95%CI 1.24–43.86, respectively]); more childhood traumas (aOR1.24, 1.11–1.38), and more patriarchal personal gender attitudes (aOR1.24, 1.00–1.54). In the oPT experiencing IPV (aOR3.07, 1.02–9.23) and borrowing and experiencing IPV (aOR5.89, 1.84–18.79) were risks for married women. For unmarried women borrowing because of hunger was associated with higher risk (aOR2.33, 95%CI 1.18–4.85). Despite limitations–specifically the potential women were reporting the same ‘honour killing’—our analysis suggests ‘honour killings’ are associated with violence, patriarchy, and poverty. Research is needed for the prevention of ‘honour killing’, which must address the root causes.

Trial Registration: ClinicalTrials.gov NCT03236948.

## Introduction

‘Honour killings’ are the most extreme form of ‘honour based abuse (HBA), which ranges from social isolation, insulting, coercion and forced marriage, and other forms of violence, all under the guise of ‘maintaining honour’ in the family [1]. Women are disproportionately the targets of HBA and ‘honour killing’, but men can also experience it. For women, forms of ‘dishonour’ include communicating with ‘unknown’ men, being in a room with a man who is not a family member, to perceived or actual adultery, running away, premarital pregnancy, or otherwise challenging patriarchal gender norms [2–4]. An estimated 5000 ‘honour killings’ occur every year [5], with the majority of these in the Middle East and Asia, and their diaspora in Europe, America and Latin America [5].

The true prevalence of ‘honour killings’ is unknown. Between March 2011 and April 2013, according to the Afghanistan Independent Human Rights Commission, 243 cases of ‘honour killings’ were registered. In just over half (56%) of cases, perpetrators were identified. Of identified perpetrators, 39% were husbands, 15% brothers, 9% fathers, 6% brothers-in-law, 5% other family members, and the remaining 26% ‘relatives’ [6]. A similar perpetrator profile is seen in an epidemiological study of ‘honour killing’ in Pakistan [4]. There is some definitional blurring, as listing husbands as perpetrators of ‘honour killings’ clearly shows overlap between ‘honour killings’ and intimate femicide. In the occupied Palestinian Territories (oPT), there is no centralized collection of data on ‘honour killings’. The Women’s Centre for Legal Aid and Counselling, reported 27 cases in 2014, and 15 in 2015 [3]. However, these small numbers may underestimate the scale of the problem and provide no basis for monitoring trends, which are perceived to have increased over the last decade [3].

Legal responses to ‘honour killings’, and HBA more widely, are often weak. In Afghanistan the 2009 Elimination of Violence Against Women Law criminalised all forms of physical, sexual and emotional violence against women, but did not include ‘honour killing’. Rather ‘honour killing’ is a common law offense, but the penal code (Article 398), provides a reduced sentence for those who kill a woman for committing adultery. Consequently, perpetrators of ‘honour killings’ often are little or un-punished [7]. In the West Bank of oPT, legal frameworks draw on the Jordanian Penal Code, including Article 340, stating if a woman commits adultery, and her husband kills her, he can be pardoned [8–10]. In the Gaza Strip, while there is no legal support for ‘honour killing’, courts often give men who have killed women because of ‘honour’ reduced sentences [3, 8].

Anthropological literature has made three main arguments about the drivers of ‘honour killing’ in communities where these are manifest. First, it is a form of patriarchal social control over women’s bodies and sexuality [2, 9, 11]. This connects ‘honour killing’ to other forms of violence against women and girls (VAWG), often noting women who are killed ‘in the name of honour’ have previously experienced other forms of violence from family members [12]. It further connects this to gender inequalities, and the dominance of male power in households and communities, and more widely other forms of HBA, including forced/coerced marriage [13, 14]. These all are shaped by similar patterns around the assertion of male patriarchal power over women in the household, although this is often upheld by women as well as men [13].

A second strand of research highlights economic factors in ‘honour killings’ [9, 11, 14]. Broadly in communities where ‘honour killings’ are more common, women’s bodies may be considered as resources for reproduction, domestic labour, and access to other resources [14]. In such contexts, arranged marriages between cousins, or other relatives, enable land and property to remain in the family [14, 15]. An online study of ‘honour based violence’ amongst Kurdish speaking people in Iraq, found it was more common for women where they had been coerced to marry their cousin [16]. ‘Honour killings’ occur partly therefore because of the dissolution and loss of control of property that is represented by the woman, and as such, they are more likely in poorer households where property is in scarcer supply [14].

A third argument suggests ‘honour’ is a resource through which families can achieve social status in a community, without recourse to economic or political routes, which for poor families is not possible [15]. As such, for poorer families, maintenance of family ‘honour’ may be more important than it is for richer families, who have multiple ways of achieving social status, and therefore ‘honour killing’ may be more common amongst poorer households.

All these arguments can be seen in a study from Jordan amongst school children of attitudes supportive of ‘honour killing’, which found these associated with the father’s use of harsh discipline against them, poverty and more conservative gender attitudes [17]. However a key question remains: in cultures where family ‘honour’ is largely read through women’s bodies, what places some families at greater risk than others of having ‘honour killings’?

The objective of this paper is to report factors associated with women reporting an ‘honour killing’ within their family in Afghanistan and the oPT. The paper has three hypotheses. First, in families that have ‘honour killings’ there is generally more VAWG, shown through women of the family being more likely to report experiencing violence from other household members including their husband. Second, women reporting ‘honour killings’ in their family are more likely to report more patriarchal gender attitudes than other women. Third, women reporting ‘honour killings’ in their family are exposed to higher levels of poverty.

## Materials and methods

Data comes from secondary analyses of two studies that were both conducted to form the baseline for separate evaluations of interventions. In the first study, between September 2016 and March 2017, 1458 women were recruited to participate in the Women for Women International (WfWI) individually randomized control trial in six villages, in two Provinces (Nangarhar and Kabul) in Afghanistan. Six villages were enrolled and community leaders approached for access. Once permission was given, we used Friday prayers, and community meetings to share information about the research and intervention, and then worked in each community for two to three days to recruit women. In each of the six villages between 114 and 413 women were enrolled [18]. Throughout, the study was described as about understanding and supporting Afghanistan and development in Afghanistan. In all villages there was a lot of interest in involvement by women [18].

Eligibility requirements were that women were aged between 18 and 49, from the poorest households and without proper work. Further eligibility criteria included residence in the village and ability to understand informed consent. At enrollment, but not questionnaire completion, participants were unaware of which study arm they were in. Those in the control arm received US$10 for questionnaire completion. Questionnaires were completed through face-to-face interviews administered by trained female fieldworkers in local languages. Participants provided informed consent to participate, marked with a thumbprint which was witnessed. Further information on study procedures are described elsewhere [18].

The second study was conducted in February 2017, in a nationally representative cross-sectional survey in the oPT. In total 354 women were enrolled. The sampling framework meant that a representative, self-weighing sample was constructed, reflecting the overall population of the oPT, based on the 2007 Population and Establishment Census (i.e. we sampled proportional to the population, and did not have to adjust the sample to make it reflective of the whole population). Random household sampling was instituted at the primary sampling unit and at household level. For inclusion, women had to be randomly selected, 18 or older, and willing to participate and provide informed consent. Female, trained enumerators, completed face-to-face interviews in Arabic, with structured questionnaires.

In both studies, data were double-entered and electronically compared. Discrepancies were compared to original documentation, and changes made before finalization of databases.

In both studies, ethical approval was received from the Research Ethics Committee of the South African Medical Research Council. In Afghanistan, the Research Ethics Committee of the Ministry of Public Health also provided ethical approval.

### Measures

Questionnaires were developed responding to contexts and the studies being conducted, and therefore differ. Wording of questions, and Cronbach’s alphas, for all questions are provided in S1 File. Variables were selected based on theoretical and hypothesized relationships.

The primary outcome in both studies was a single item: “Has any girl or woman relative been killed in the name of honour by members of your family?” Responses were yes or no. For women reporting ‘yes’ they were asked about how recent the most recent murder was, with responses: less than one year, one to five years, or six and more years ago. We recognize that in Afghanistan and the oPT ‘family’ is a broader term than nuclear family, and can encompass extended family members and tribal relationships too, and that our sampling strategy may have led to more than one person reporting the same ‘honor killing’.

Socio-demographics were asked of all women. We asked about women’s age, highest level of education, marital status (never married, currently married, and divorced/widowed), and, if married, whether they were previously related to their husband (no, cousin, other relative).

In both studies a single item assessed resource mobilisation (ease of accessing cash in an emergency), which is typically an indicator of poverty, but also reflects strength of social networks, pertinent here are family ties. In Afghanistan poverty was assessed through an item asking whether women had borrowed money or food in the past month because of hunger (More than once, or almost every week, versus once or twice a month or never), and through three items assessing household level food-insecurity [19].

Currently married women were asked five behaviourally-specific questions about past year experiences of physical IPV in both studies. These were based on the WHO Multi-Country Study on Domestic Violence [20], and previously used across Asia-Pacific and the Middle East [21]. A positive response to at least one item led women being coded as experiencing past year physical IPV. Married Afghan women were also asked whether their mother-in-law had hit them in the past year. We also asked married Afghan women seven questions about her relationship to her husband (e.g. My husband is a kind person), with responses on a Likert Scale: Strongly Agree; Agree; Disagree; and Strongly Disagree. We summed these items and higher scores represented a worse relationship with husband.

All Afghan women were asked about other forms of familial violence, including whether they had been hit by a parent, and separately by their sibling, in the past year. Women’s childhood traumas were assessed using 12 items based on childhood trauma scales [22] adapted for Afghanistan, and summed. Items included experiences of physical violence, hunger, and neglect before the age of 18.

Women’s own gender attitudes were assessed. In Afghanistan an 11 item, locally developed scale was constructed [18], while in the oPT seven items were based on the GEM scale [23]. In both scales, items asked about personal views around gender norms and roles, and responses were on a four-point Likert scale, and items summed with higher scores indicating more patriarchal gender attitudes.

### Analysis

Data from both countries were uploaded and compiled into two separate datasets for analysis in Stata/IC 14.1. All analyses were by country. We summarized the data for age, education, marital status and province (or location in oPT) as well as proportion of the participants reporting an ‘honour killing’ in their family and how recent the last ‘honour killing’ was using percentages and 95% confidence intervals (CIs), calculated to take into account the design of the studies, using Taylor linearization.

To describe associations between hypothesized independent variables and reporting an ‘honour killing’, we calculated the proportion (or mean score) of women per category of each independent variable who had, and who had not, reported an ‘honour killing’ in the family. We calculated 95% CIs using Taylor linearization [24] and tested for association using a Chi squared test. For continuous variables we compared mean scores using an Adjusted Wald test reporting 95% CIs and p-values. The analysis of past year IPV and beating by a mother-in-law were restricted to currently married women.

We built separate logistic regression models for ‘honour killing’ for married women, and unmarried women, by country. The models for married women enabled the testing of variables which were only responded to by married women (specifically IPV and violence from mother-in-laws). Logistic regression models included all hypothesized variables. The models were built with no elimination of variables. Based on recommendations from the American Statistical Association we report adjusted odds ratios (aOR) and 95%CI [25].

Because of the relatively small sample size, we addressed interactions between variables by developing and testing model-appropriate dummy variables, for each of the four sub-groups (currently married/not married and oPT/Afghanistan). For married women in Afghanistan, there was an interaction between abuse by husband and by mother-in-law. We developed a four level variable: no violence; only mother-in-law violence; only IPV; and both forms of violence. For unmarried Afghan women, there was interaction between abuse by a parent, and by a sibling, and we developed a three-level variable: no violence; only one form of abuse (either partner or sibling); both forms of violence. For married women in oPT there was interaction between borrowing money or food in the last month and experience of IPV. We derived a four level variable with levels being: neither, borrowing money but not experiencing IPV; just experiencing IPV; and both.

## Results and discussion

### Afghanistan

The sample in Afghanistan comprised 1461 women (Table 1). Half (50.8%) the women were aged 18–29, a third (28.5%) 30–39 and a fifth (20.7%) were forty or older. Over three-quarters (77.1%) had no education. Almost two-thirds (64.0%) were currently married, only 6.7% were divorced or widowed, and a third (29.3%) were unmarried. Three-quarters (75.4%) came from Kabul and a quarter Nangarhar Province.

**Table 1:** Descriptive statistics for samples in Afghanistan and the occupied Palestinian Territories.

	Afghanistan			occupied Palestinian Territories		
Socio-demographics	N	n	%(95%CI)	N	n	%(95%CI)
***Age Group***	1461			535		
18–29		742	50.8(48.2–53.4)		212	39.6(35.6–43.8)
30–39		417	28.5(26.3–30.9)		127	23.7(20.3–27.6)
40+		302	20.7(18.7–22.8)		196	36.6(32.7–40.8)
***Education***	1457			535		
None		1124	77.1(75.0–79.2)		10	1.9(1.0–3.4)
Primary, secondary or more		333	22.9(20.8–25.0)		525	98.1(96.6–98.9)
***Marital status***	1461			535		
Currently married		935	64.0(61.5–66.4)		366	68.4(64.5–72.1)
Divorced or widowed		98	6.7(5.5–8.1)		46	8.6(6.5–11.3)
Unmarried		428	29.3(27.0–31.7)		123	23.0(19.7–26.7)
***Palestinian Territory***				535		
Gaza					200	37.4(37.2–37.6)
West Bank					335	62.6(62.4–62.9)
***Afghanistan Province***	1461					
Kabul		1101	75.4(75.4–75.4)			
Nangarhar		360	24.6(24.6–24.6)			
**‘Honour’ Killing**						
Ever experienced ‘honor killing’ in family	1460	33	2.3(1.6–3.2)	535	41	7.7(5.8–10.1)
***Recentness of ‘honor killing’***	32			39		
Past year		11	34.4(19.5–53.1)		8	20.5(11.4–34.1)
1–5 years ago		12	37.5(22.0–56.1)		12	30.8(22.4–40.7)
6 or more years ago		9	28.1(14.8–46.9)		19	48.7(40.8–56.7)

Among all Afghan women, 2.3% (n = 33) of women disclosed that there had been an ‘honour killing’ in their family. Of these, a third (34.4%) said it occurred in the past year, another third one to five years ago (37.5%) and the rest six or more years ago.

In bivariate analysis (Table 2) the proportion of ‘honour killings’ was higher amongst women from Nangarhar (66.7%) compared to Kabul Province (23.7%), those reporting borrowing in the past month because of poverty (45.5%) compared to those not (22.5%, p = 0.002), and women reporting easier access to money in an emergency (39.4%) compared to those who would find it hard (22.5%, p = 0.001). Amongst married women, worse relationship (higher) mean scores were reported by women who reported an ‘honor killing’ in the family (p<0.001). A higher proportion of married women who reported past year physical IPV (54.6%) compared to those who did not (22.4%, p = 0.004), reported an ‘honour killing’, as did a higher proportion reporting past year violence from a mother-in-law (38.1%) compared to those who did not (22.4%, p = 0.021). Similarly, amongst all Afghan women, a higher proportion of Afghan women reporting an ‘honour killing’ reported being beaten by a parent (48.5% c.f. 13.2%, p<0.001) or sibling (33.3%, c.f. 12.9%, p = 0.007), and they disclosed more childhood traumas (p = 0.0263). Women reporting an ‘honour killing’ had more patriarchal gender attitudes (p = 0.066).

**Table 2:** Descriptive associations between ‘honour killing’ and socio-demographic, poverty, other forms of violence, and gender attitudes in Afghanistan and the occupied Palestinian Territories.

	Afghanistan						occupied Palestinian Territories					
	No ‘honour killing’ reported in family			‘Honor killing’ reported in family			No ‘honour killing’ reported in family			‘Honor killing’ reported in family		
Socio-demographics	N	n	%/mean(95%CI)	n	%/mean(95%CI)	pvalue	N	n	%/mean(95%CI)	n	%/mean(95%CI)	pvalue
***Age Group***												
18–29	740	720	50.5(47.9–53.1)	20	60.6(43.3–75.6)		212	195	39.5(35.3–43.8)	17	41.5(27.9–56.4)	
30–39	417	408	28.6(26.4–31.0)	9	27.3(14.8–44.7)		127	122	24.7(21.1–28.7)	5	12.2(5.2–26.1)	
40+	301	297	20.8(18.8–23.0)	4	12.1(4.6–28.2)	0.396	196	177	35.8(31.7–40.2)	19	(46.3(31.9–61.4)	0.155
***Education***												
None		1095	77.2(74.9–79.3)	25	75.8(58.6–87.4)		10	5	1.0(0.4–2.4)	5	12.2(5.4–25.5)	
Primary, secondary or more		324	22.8(20.8–25.1)	8	24.2(12.6–41.4)	0.848	525	489	99.0(97.6–99.6)	36	87.8(74.6–94.7)	<0.001
***Palestinian territories***												
Gaza							200	182	36.8(35.7–38.0)	18	43.9(30.8–57.9)	
West Bank							335	312	63.2(62.0–64.3)	23	56.1(42.1–69.2)	0.345
***Afghanistan—Province***												
Kabul	1099	1088	76.4(75.9–76.8)	11	33.3(19.7–50.6)							
Nangarhar	359	337	23.7(23.2–24.1)	22	66.7(49.5–80.4)	<0.001						
**Poverty**												
Borrowed in past month because of hunger (yes)	336	321	22.5(20.5–24.7)	15	45.5(29.6–62.3)	0.002	106	91	18.4(15.5–21.8)	15	36.6(23.1–52.6)	0.006
Access to money in emergency (easy)	262	249	17.5(15.6–19.5)	13	39.4(24.5–56.6)	0.001	250	235	47.6(43.7–51.5)	15	36.6(23.3–52.2)	0.18
***Marital status***												
Currently married	932	910	63.9(61.4–66.3)	22	66.7(49.2–80.5)		366	340	68.8(64.7–72.7)	26	63.4(48.4–76.2)	
Divorced or widowed	98	98	6.9(5.7–8.3)	0	0(0.0–0.0)		46	42	8.5(6.4–11.3)	4	9.8(3.7–23.5)	
Never married	428	417	29.3(27.0–31.2)	11	33.3(19.5–50.8)	0.281	123	112	22.7(19.2–26.6)	11	26.8(15.7–42.0)	0.768
***Is husband a relative*?**												
No	372	363	40.0(37.0–43.2)	9	40.9(22.8–61.9)		245	231	60.5(55.7–65.1)	14	46.7(29.5–64.7)	
Cousin	431	420	46.3(43.1–49.5)	11	50.0(30.2–70.0)		84	79	20.7(17.0–25.0)	5	16.7(7.2–34.2)	
Other relative	126	124	13.7(11.6–16.0)	2	9.1(2.3–30.1)	0.82	83	72	18.9(15.3–23.0)	11	36.7(21.4–55.2)	0.069
Relationship with husband (> = worse)	932		14.8(14.6–14.9)		16.8(15.7–17.9)	<0.001						
**Violence in household**												
Past year physical IPV (yes)	215	203	22.4(19.8–25.1)	12	54.6(34.1–73.6)	<0.001	93	81	23.8(20.0–28.1)	12	46.2(28.3–65.1)	0.014
Past year hit by mother-in-law (yes)	117	109	18.1(15.3–21.3)	8	38.1(20.3–59.8)	0.021						
Parent hit them in past year (yes)	200	184	13.2(11.5–15.0)	16	48.5(32.3–65.0)	<0.001						
Hit by sibling in past year (yes)	193	182	12.9(11.3–14.8)	11	33.3(19.5–50.7)	<0.001						
Childhood traumas > = more	1450		15.5(15.3–15.7)		17.8(15.8–19.8)	0.026						
**Attitudes**												
Individual gender attitudes (> = more patriarchal)	1457		22.15(21.99–22.31)		23.18(22.10–24.27)	0.066	518		10.5(10.3–10.7)		11.4(10.5–12.2)	0.069

In multivariable logistic regression model for married women in Afghanistan (Table 3) ‘honour killing’ was associated with borrowing in the past month because of hunger (aOR8.71, 95%CI 2.27–33.40), and households finding it easier to access money in an emergency (aOR11.39, 95%CI 3.05–42.50). Additionally, ‘honor killing’ was associated with reporting only experiencing IPV (aOR3.73, 95%CI 1.12–12.36), and those reporting experiencing IPV and violence from their mother-in-law (aOR10.52, 95%CI 2.60–42.56). There was some evidence that married women reporting an ‘honour killing’ had had less childhood trauma than those who did not (aOR0.86, 95%CI 0.75–1.00).

**Table 3:** Factors associated with married women reporting ‘honour killing’ in Afghanistan (n = 899).

	aOR	95%CI
Province (Nangarhar c.f. Kabul)	2.04	0.73–5.68
Age (continuous)	0.95	0.89–1.01
Borrowed in past month because of hunger (yes)	8.71	2.27–33.40
Ease of accessing money in an emergency (easy)	11.39	3.05–42.50
Relationship with husband (> = worse)	0.61	0.23–1.67
***IPV and MIL violence***		
No IPV or mother-in-law violence	ref	
Mother-in-law violence only, no IPV	1.05	0.19–5.88
Only IPV, but no mother-in-law violence	3.73	1.12–12.36
IPV and mother-in-law violence	10.52	2.60–42.56
Childhood traumas (> = more)	0.86	0.75–1.00

Chi-square <0.00001, R2 = 0.25

Multivariate logistic regression for unmarried Afghan women is presented in Table 4. In this analysis, ‘honour killings’ were associated with a household finding it easier to access money in an emergency (aOR4.06, 95%CI 0.85–19.37). In addition, women who reported experiencing both parental and sibling violence in the past year (aOR1.24–43.86), more childhood traumas (aOR1.24, 95%CI 1.11–1.38), and having more patriarchal individual gender attitudes (aOR1.24, 95%CI 1.00–1.54), also were more likely to report ‘honour killing’ in the household.

**Table 4:** Factors associated with ‘honour killing’ in the family amongst unmarried women in Afghanistan (n = 502).

	aOR	95%CI
Ease of accessing money in an emergency (easy)	4.06	0.85–19.37
***Parental/sibling violence***		
None	ref	
Parental or sibling violence	5.47	0.82–36.70
Parental and sibling violence	7.37	1.24–43.86
Childhood traumas (> = more)	1.24	1.11–1.38
Individual gender attitudes (> = more patriarchal)	1.24	1.00–1.54

Chi squared <0.00001, R2 = 0.30

### The occupied Palestinian Territories

In the oPT, 535 women were interviewed. A third (39.8%) were aged 18–29, a quarter (23.7%) were 30–39 and a third (36.6%) were 40 or older. Only 1.9% reported no education. Two-thirds of women (68.4%) were currently married, 8.6% divorced or widowed, and a quarter (23.0%) unmarried. Two-thirds (62.6%) were from the West Bank and a third (37.4%) from Gaza. Amongst the whole sample, 7.7% (n = 41) of women reported an ‘honour killing’ in their family. A fifth (20.5%) said it was in the past year, a third (30.8%) 1–5 years previously, and half (48.7%) six or more years ago.

In bivariate analysis (Table 2) a larger proportion of women with no education (99.0%) (compared to any education 87.8%, p<0.00001), those who reported borrowing in the past month because of hunger (36.6%, compared to 18.4%, p = 0.006), and those reporting more patriarchal gender attitudes (p = 0.069) reported an ‘honour killing’. Amongst married women a greater proportion of women who were married to a relative which was not their cousin (36.7%, c.f. 18.9%) and a greater proportion of women reporting past year IPV (46.2%, c.f. 23.8%, p = 0.014) reported an ‘honour killing’ in their family ever.

Table 5 reports the multivariable logistic regression model married women in the oPT. Women reporting having any education were less likely to report ‘honour killing’ (aOR0.05, 0.01–0.19) than those with no education. Compared to those reporting no borrowing and no IPV, those reporting borrowing in the past month but no IPV (aOR2.77, 95%CI 0.94–8.09), IPV (but not borrowing) (aOR3.07, 95%CI 1.02–9.23), and those reporting borrowing and IPV (aOR5.89, 95%CI 1.84–18.79), were all more likely to report an ‘honor killing’. Women who were married to a relative (but not a cousin) were also more likely to report ‘honor killing’ (aOR3.30, 95%CI 1.34–8.15).

**Table 5:** Factors associated with ‘honour killing’ amongst married women in the occupied Palestinian Territories (n = 412).

	aOR	95%CI
Education (primary or more)	0.05	0.01–0.19
***Husband related to you***		
No	ref	
Cousin	1.04	0.32–3.35
Other relative	3.30	1.34–8.15
***Borrowing and IPV***		
No borrowing or IPV	ref	
Only borrowed in past month, no IPV	2.76	0.94–8.09
Only IPV, no borrowing	3.07	1.02–9.23
Borrowed and IPV	5.89	1.84–18.79
Refugee camp (c.f. other settlements)	2.17	0.94–5.03

Chi-square <0.00001, R2 = 0.16

Amongst unmarried women in the oPT (Table 6) the multivariable logistic regression model showed education being protective of reporting an ‘honour killing’ (aOR0.15, 95%CI 0.02–0.86) and there was an association between borrowing in the past month and ‘honour killing’, whereby those reporting borrowing were more likely to report an ‘honour killing’ (aOR2.33, 95%CI 1.18–4.85), compared to those reporting no borrowing.

**Table 6:** Factors associated with ‘honour killing’ amongst unmarried women in the occupied Palestinian Territories (n = 160).

	aOR	95%CI
Education (none)	ref	
Education (primary or more)	0.08	0.02–0.29
Borrowing in past month because of hunger (no)	ref	
Borrowing in past month because of hunger (yes)	2.33	1.18–4.85
Gender attitudes (> = more patriarchal)	1.06	0.94–1.19

Chi-square p<0.0001, R2 = 0.07

## Discussion

This research suggests ‘honour killings’ may be common in these two countries. In the generalisable, population-based sample from oPT, 1 in 13 women in the population of West Bank and Gaza lived in a family where there had been an ‘honour killing’. In Afghanistan, 1 in 16 women interviewed from Nangarhar, and 1 in 100 from Kabul, provinces reported an ‘honour killing’ in their family. This may, however, be an overestimate as a number of women may be reporting the same ‘honour killing’ because of the sampling strategy, and we did not clarify women’s relationship to the murdered woman. As such, while it is not possible to derive population prevalence from this data it does suggest the problem maybe more common than suggested by estimates from Non-Governmental Organisations and the Afghan Human Rights Commission [6, 8].

As hypothesized, our analysis suggests a strong association between women reporting ‘honour killing’ in their family and reporting other violence from household members, suggesting a clustering of violent practices within families. Furthermore, the analysis also highlights an association between poverty and ‘honour killings’. Despite there being a significant body of qualitative research on ‘honour killings’ [2, 9, 11, 12, 15], these findings provide rare quantitative evidence supporting ethnographic research on the root causes of ‘honour killings’.

The consistent association in the multivariable regression analyses across both countries between other forms of violence in the home, including violence from a husband, beating from a parent, or mother-in-law, childhood traumas, and sibling violence, strongly supports an interpretation that ‘honour killing’ is an extension of other forms of VAWG and can be understood as an overall pattern of control and violence directed at women and girls by men, to control women’s mobility, autonomy and sexuality. Previous quantitative work has demonstrated this overlap in relation to other forms of family violence, including IPV and violence against children [26], and IPV and mother-in-law violence [27, 28], and it has been argued that family dynamics sustain and enable these overlaps [26, 28, 29]. Qualitative studies have also shown patterns of family violence occurring prior to an ‘honour killing’ [11, 12], reflecting the femicide literature, which suggests the murder of women is the culmination of other forms of violence perpetrated by men and experienced by women [30, 31]. Similarly, research on HBA has also emphasized the multitude of perpetrators, including mother-in-laws and others [13], reflecting household acceptability of violence as very important in shaping HBA, and ‘honor killing’.

The analysis suggests an unclear association between gender attitudes (as measured) and ‘honour killing’. The use of violence is a strongly gendered practice, and when directed towards women and girls within families is clearly an expression of patriarchal gender norms [5, 21]. As the analysis highlighted, there was an important clustering of violent practices within the household. However, in adjusted models, individual patriarchal gender attitudes were only associated with ‘honour killing’ amongst unmarried women in Afghanistan. This lack of association may be because our scale did not adequately capture how gender attitudes are articulated in these settings, and how patriarchy is enforced. More widely, however, anthropological literature has emphasised that the family has remained particularly important in shaping gender relationships in Afghanistan and the oPT because of ongoing conflict and occupation [2, 32], the relative lack of state power and, the reliance on agriculture [15].

There were complex associations between poverty and ‘honour killing’. In Afghanistan and the oPT, women reporting borrowing more in the past four weeks because of hunger, were more likely to report an ‘honour killing’ in the family. Borrowing may be an indication of poverty, and women who borrow more, may be perceived as a burden on a family. This supports the anthropological literature [15] that poverty drives ‘honour killing’, although the precise reasons why are not clear. Previous research has similarly highlighted that other forms of VAW are connected to poverty [33].

In Afghanistan, women reporting easier to access money in an emergency were more likely to report an ‘honour killing’, but this was not the case in the oPT. Ease in accessing money in an emergency has been conceptualized as a measure of wealth/poverty [33], and we had hypothesized that challenges in accessing money in an emergency would be associated with ‘honour killing’. However, in contexts where formal banking systems do not commonly exist, access to money in an emergency may also be an indicator of the strength of family and social networks. Anthropological literature highlights the importance of establishing support for an ‘honour killing’ from other community members and institutions [11, 15], suggesting strong networks facilitate this. It may also be that tighter extended family networks, ensure knowledge of ‘honour killings’ is also communicated more widely to the extended family.

The lack of association in the oPT between ease of accessing money and ‘honour killing’ may be because women who report borrowing are poorer and less educated. Access to resources is deceiving; as it really indicated more reliance/ dependency on family, and as such more vulnerability to family whims. Many of the women who borrow are more perceived as a burden on the family, rather than an indication of (positive) family ties. However, qualitative work would have to untangle these associations fully.

In the oPT education (primary or more) was associated with less ‘honour killing’ in the family. Previous research has suggested education is associated with attitudes to ‘honour killing’ and people with more education have less patriarchal attitudes [17, 21]. In contexts with relatively high levels of women’s education, lack of education may be indicative of social marginalization, and a proxy for other forms of poverty. In Afghanistan so few women had more than madrassa or basic primary education, and women’s attending education is so contested and closely related to household gender attitudes that the measure would not have been expected to operate in the same way as in oPT.

The analysis has a number of implications for future research and intervention work on violence against women and girls. First, recognising ‘honour killing’ as one important form of femicide and asking about it, alongside other forms of femicide (e.g. intimate femicide) is crucial for understanding the magnitude of violence women experience. Future research should ask more directly about the relationship between interviewee and the woman killed, and explore how this violence relates to other patterns of women experience. Given there appears to be overlapping associations between other forms of violence women experience (IPV, mother-in-law violence etc.) interventions working to eradicate violence against women, may find it appropriate to include work around ‘honour killing’. The close connections between all forms of family violence and ‘honour killing’ do suggest that prevention efforts be targeted at working with families around preventing violence more generally, as well as ‘honour killing’ specifically.

This study has a number of limitations. The main limitation is we are unsure of the relationship between the participant and the woman or girl killed in the name of ‘honour’. The term family extends beyond nuclear families, to include extended families, and potentially tribal networks. As such, we cannot be sure that some women were not reporting the same ‘honour killing’ and so, even in oPT, cannot use the data to derive a prevalence of ‘honour killings’. In addition, questions about violence, poverty and gender attitudes were all within the past year, but we cannot be sure when the person was killed. However, it is unlikely that personal ideas on gender relations would have become more conservative and to the extent that these reflect social norms in the family it is more likely that these would not have changed much over time. Both samples were cross-sectional and as such temporality of associations are unclear. We only had one marker of poverty in oPT, and the item could have multiple meanings. In Afghanistan, women self-selected into the study, therefore results are not generalizable to the population level, but this does not necessarily invalidate associations. To the extent women are highly constrained in Afghanistan, we would expect trial participants to be likely to come from relatively less controlling families. Finally, the proportion of women reporting an ‘honour killing’ was low, resulting in limited power to find associations in analysis.

## Conclusions

‘Honour killings’ are not isolated incidents but are probably much more common than is normally estimated. They should be documented and researched as part of strategies to eradicate them. As we hypothesized, in these countries they were associated with other forms of VAWG and poverty. While ‘honour killings’ are distinctive among other types of female murder, because of the perpetrators and ‘expressed’ reasons, the killing of women is located within, and driven by, similar practices of control of women’s sexuality and enforcement of male power. Prevention of ‘honour killings’ needs to be an explicit development goal, and achieved through changing patriarchal gender norms, reducing poverty and removing the impunity of perpetrators.

## Supporting information

S1 File Questionnaires used on the two studies. (DOCX)

## References

[1] Khan R. Introduction to the special issue on honour-based abuse, violence and killings. Journal of Aggression, Conflict and Peace Research. 2018;10(4):237–8.

[2] Ruggi S. Commodifying honor in female sexuality: Honor killings in Palestine. Middle East Report. 1998:12–5.

[3] Special Rapporteur on Violence Against Women. Report of the Special Rapporteur on violence against women, its causes and consequences, on her mission to the Occupied Palestinian Territory/State of Palestine. New York: Human Rights Council, 2017.

[4] Nasrullah M, Haqqi S, Cummings KJ. The epidemiological patterns of honour killing of women in Pakistan. European Journal of Public Health. 2009;19(2):193–7. 10.1093/eurpub/ckp021 19286837

[5] WHO. Understanding and Addressing Violence Against Women: Femicide. Geneva: WHO, 2012.

[6] Afghanistan Independent Human Rights Commission. National Inquiry on Rape and Honor Killing in Afghanistan Report. Kabul: Afghanistan Independent Human Rights Commission, 2013.

[7] United Nations Assistance Mission in Afghanistan, Office of the United Nations High Commissioner for Human Rights. Injustice and Impunity: Mediation of Criminal Offences of Violence against Women. Kabul, Afghanistan: UNAMA/OHCHR, 2018.

[8] Al Ashqar A. Murder of Women in Palestine under the Pretext of Honour: Legislation and Jurisprudence Analytical Study. Ocuppied Palestinian Territories: United Nations Human Rights, 2014.

[9] Said-Foqahaa N, Maziad M. Arab women: Duality of deprivation in decision-making under patriarchal authority. Hawwa. 2011;9(1):234–72.

[10] Said N, Amasheh M, Said S, Ghattas R, Wright K, Shaui’bi M, et al. Caught up between a Rock & a Hard Place: Occupation, Patriarchy and Gender Relations, (A Case Study of Palestinian Women in Area C & H2). Palestine: EU, UN Women, AWRAD, 2018.

[11] Sev’er A, Yurdakul G. Culture of honor, culture of change: A feminist analysis of honor killings in rural Turkey. Violence against women. 2001;7(9):964–98.

[12] Payton J. “Honor,” Collectivity, and Agnation: Emerging Risk Factors in “Honor”-Based Violence. J Interpers Violence. 2014;29(16):2863–83. 10.1177/0886260514527171 24671044

[13] Aplin R, editor Exploring the role of mothers in ‘honour’based abuse perpetration and the impact on the policing response Women’s Studies International Forum; 2017: Elsevier.

[14] Mayeda DT, Vijaykumar R. A Review of the Literature on Honor‐based Violence. Sociology Compass. 2016;10(5):353–63.

[15] Awwad AM. Gossip, scandal, shame and honor killing: A case for social constructionism and hegemonic discourse. Social thought & research. 2001:39–52.

[16] Payton JL. For the Boys in the Family: An Investigation Into the Relationship Between “Honor”-Based Violence and Endogamy. J Interpers Violence. 2017;32(9):1332–57. 10.1177/0886260515588918 26048164

[17] Eisner M, Ghuneim L. Honor killing attitudes amongst adolescents in Amman, Jordan. Aggressive behavior. 2013;39(5):405–17. 10.1002/ab.21485 23744567

[18] Gibbs A, Corboz J, Shafiq M, Marofi F, Mecagni A, Mann C, et al An individually randomized controlled trial to determine the effectiveness of the Women for Women International Programme in reducing intimate partner violence and strengthening livelihoods amongst women in Afghanistan: trial design, methods and baseline findings. BMC Public Health. 2018;18(1):164 10.1186/s12889-018-5029-1 29357843 PMC5778673

[19] Coates J, Swindale A, Bilinsky P. Household Food Insecurity Access Scale (HFIAS) for measurement of food access: indicator guide. Washington, DC: Food and Nutrition Technical Assistance Project, Academy for Educational Development 2007.

[20] Garcia-Moreno C, Jansen HAFM, Ellsberg M, Heise L, Watts CH, Wo WM-CS. Prevalence of intimate partner violence: findings from the WHO multi-country study on women’s health and domestic violence. Lancet. 2006;368(9543):1260–9. 10.1016/S0140-6736(06)69523-8 17027732

[21] Fulu E, Miedema S, Roselli T, McCook S, Chan KL, Haardörfer R, et al Pathways between childhood trauma, intimate partner violence, and harsh parenting: findings from the UN Multi-country Study on Men and Violence in Asia and the Pacific. The Lancet Global Health. 2017;5(5):e512–e22. 10.1016/S2214-109X(17)30103-1 28395846

[22] Bernstein DP, Stein JA, Newcomb MD, Walker E, Pogge D, Ahluvalia T, et al Development and validation of a brief screening version of the Childhood Trauma Questionnaire. Child abuse & neglect. 2003;27(2):169–90. 12615092 10.1016/s0145-2134(02)00541-0

[23] Pulerwitz J, Barker G. Measuring attitudes toward gender norms among young men in Brazil development and psychometric evaluation of the GEM scale. Men and Masculinities. 2008;10(3):322–38.

[24] Paben S, editor Comparison of Variance Estimation Methods for the National Compensation Survey. Proceedings of the Section on Survey Research Methods, American Statistical Association; 1999.

[25] Wasserstein RL, Lazar NA. The ASA’s statement on p-values: context, process, and purpose. The American Statistician. 2016;70(2):129–33.

[26] Namy S, Carlson C, O’Hara K, Nakuti J, Bukuluki P, Lwanyaaga J, et al Towards a feminist understanding of intersecting violence against women and children in the family. Social Science & Medicine. 2017;184:40–8. 28501019 10.1016/j.socscimed.2017.04.042 PMC5737762

[27] Gibbs A, Corboz J, Jewkes R. Factors associated with recent IPV experience amongst currently married women in Afghanistan and health impacts of IPV: a cross sectional study. BMC Public Health. 2018. 10.1186/s12889-018-5507-5 PMC5934802 29724222

[28] Clark CJ, Silverman JG, Shahrouri M, Everson-Rose S, Groce N. The role of the extended family in women’s risk of intimate partner violence in Jordan. Social Science & Medicine. 2010;70(1):144–51. 19837499 10.1016/j.socscimed.2009.09.024

[29] Naved RT, Rahman T, Willan S, Jewkes R, Gibbs A. Female garment workers’ experiences of violence in their homes and workplaces in Bangladesh: a qualitative study. Soc Sci Med. 2018;196:150–7. 10.1016/j.socscimed.2017.11.040 29182963

[30] Mathews S, Jewkes R, Abrahams N. ‘I Had a Hard Life’: Exploring Childhood Adversity in the Shaping of Masculinities among Men Who Killed an Intimate Partner in South Africa. British Journal of Criminology. 2011;51(6):960–77.

[31] Abrahams N, Jewkes R, Martin LJ, Mathews S, Vetten L, Lombard C. Mortality of women from intimate partner violence in South Africa: a national epidemiological study. Violence and victims. 2009;24(4):546 19694357 10.1891/0886-6708.24.4.546

[32] Shalhoub-Kervorkian N, Daher-Nashif S. Femicide and colonization: between the politics of exclusion and the culture of control. Violence against women. 2013;19(3):295–315. 10.1177/1077801213485548 23676446

[33] Jewkes R, Fulu E, Naved RT, Chirwa E, Dunkle K, Haardörfer R, et al Women’s and men’s reports of past-year prevalence of intimate partner violence and rape and women’s risk factors for intimate partner violence: A multicountry cross-sectional study in Asia and the Pacific. PLoS medicine. 2017;14(9):e1002381 10.1371/journal.pmed.1002381 28873087 PMC5584751

