# Supplementary material for: Factors associated with ‘honour killing’ in Afghanistan and the occupied Palestinian Territories: Two cross-sectional studies
Source: PLoS One. 2019 Aug 8;14(8):e0219125. doi: 10.1371/journal.pone.0219125 (PMC6687286; doi:10.1371/journal.pone.0219125)
Supplement: S1 File — (DOCX) [file pone.0219125.s001.docx]

**Supplementary File 1. Questionnaires used on the two studies**

|  |  |  | Afghanistan (Cronbach alpha) | Occupied Palestinian Territories (Cronbach alpha) |
| --- | --- | --- | --- | --- |
| Number items | Questions | Responses (Coding in analysis) |  |  |
| Measures of poverty | | | | |
| 1 | If you had an emergency at home and needed 500 Afghani/ NIS50 (oPT), how easy would you say it would be to find the money? | Very difficult, somewhat difficult, versus fairly easy, easy  (very difficult/somewhat difficult v fairly easy/easy) | x | x |
| 1 | How often in the past 4 weeks have you had to borrow food or money because you did not have enough? | More than once a week; almost every week; once or twice a month; never  (more than once a week/almost every week v once or twice a month/never) | x |  |
| Past 12 month physical IPV (married women only) | | | | |
| 5 | In the past 12 months how many times has your husband slapped you or thrown something at you which could hurt you?  In the past 12 months how many times has your husband pushed or shoved you?  In the past 12 months how many times has your husband hit you with a fist or with something else which could hurt you?  In the past 12 months, how many times has your husband kicked, dragged, beaten, choked or burnt you?  In the past 12 months, how many times has your husband threatened to use or actually used a gun, knife or other weapon against you? | Never; once; few; many  (Never v once/few/many) | x | x |
| Relationship with husband (married women only) | | | | |
|  | My husband does not really understand me.  My husband does everything he can to support me.  My husband is a kind person.  My husband shows me he loves me often  My husband is very strict and controlling.  My husband can be cruel  My husband can frighten me | Strongly disagree; disagree; agree; strongly agree | x (0.79) |  |
| Family violence | | | | |
| 1 | In the last 12 months were you slapped, hit or beaten by your mother-in-law? (Married women only) | Never; sometimes; often  (Never v sometimes/often) | x |  |
| 1 | In the last 12 months were you slapped, hit or beaten by your mother or father? | Never; sometimes; often  (Never v sometimes/often) | x |  |
| 1 | In the last 12 months were you slapped, hit or beaten by your brother or sister or a brother or sister or other relative of your husband? | Never; sometimes; often  (Never v sometimes/often) | x |  |
| Childhood traumas | | | | |
| 8 | Before I married I saw or heard my mother being beaten by her husband  Before I married I saw or heard my mother being beaten by my mother-in-law or another person in the family  Before I married I was beaten at home with a belt or stick or whip or something else which was hard  Before I married I was beaten so hard at home that it left a mark or injured me  Before I married I was told I was lazy or stupid or weak by someone in my family  Before I married I was insulted or humiliated by someone in my family in front of other people  Before I married one or both of my parents was not able to take care of me  Before I married I was able to spend time outside the home in fields or in the garden or orchard | Never; once; few; many | x (0.65) |  |
| Gender attitudes | | | | |
| 11 | I think girls in my family should go to school  I think the husbands in my family should give permission to give their wives to go to the clinic  I think the husbands in my family should listen to their wives’ opinion on schooling  I think the wives in my family should have a say in how money is spent  I think the wives in my family should be able to ask a religious scholar about religious issues  I think the husbands in my family should respect the opinion of their wives on matters related to income generating work  I think husbands in my family should be kind and care about the happiness of women in the family  I think that the wives in my family should always obey their husbands  I think that if a wife in my family does something wrong her husband has the right to punish her  I think it is a good thing for a young wife in my family to be beaten to teach her how to behave properly  I think that a wife in my family who does things that are wrong should be beaten to correct her behavior | Strongly disagree; disagree; agree; strongly agree | x (0.87) |  |
| 7 | I think that a woman should obey her husband  I think that a man should have the final say in all family matters  I think that men should share the work around the house with women such as doing dishes, cleaning and cooking  I think that a woman cannot refuse to have sex with her husband.  I think that if a wife does something wrong her husband has the right to punish her  I think that when women work they are taking jobs away from men.  I think that there are times when a woman deserves to be beaten. | Strongly disagree; disagree; agree; strongly agree |  | x (0.63) |
